# Force-Induced Visualization of Nucleic Acid Functions with Single-Nucleotide Resolution

**DOI:** 10.3390/s23187762

**Published:** 2023-09-08

**Authors:** Qiongzheng Hu, Haina Jia, Yuhong Wang, Shoujun Xu

**Affiliations:** 1 Department of Chemistry, University of Houston, Houston, TX 77204, USA; huqz@qlu.edu.cn (Q.H.);; 2 Department of Biology and Biochemistry, University of Houston, Houston, TX 77204, USA; ywang60@uh.edu

**Keywords:** nucleic acid, drug screening, ribosome translocation, biological sensing, mechanical force

## Abstract

Nucleic acids are major targets for molecular sensing because of their wide involvement in biological functions. Determining their presence, movement, and binding specificity is thus well pursued. However, many current techniques are usually sophisticated, expensive, and often lack single-nucleotide resolution. In this paper, we report the force-induced visualization method that relies on the novel concept of mechanical force to determine the functional positions of nucleic acids with single-nucleotide resolution. The use of an adjustable mechanical force overcomes the variation of analyte concentration and differences in buffer conditions that are common in biological settings. Two examples are described to validate the method: one is probing the mRNA movement during ribosomal translocation, and the other is revealing the interacting sites and strengths of DNA-binding drugs based on the force amplitude. The flexibility of the method, simplicity of the associated device, and capability of multiplexed detection will potentially enable a broad range of biomedical applications.

## 1. Introduction

Nucleic acids are among the most important and frequent molecular targets for the field of chemical sensing, with applications ranging from virus detection to fundamental biological research [1,2,3,4]. Determining their exact sequence and interaction sites with other biological entities is usually required to understand their roles in diverse functions. Examples include molecular movement along the nucleic acids and site-specific interactions with biomolecules and drugs [5,6,7]. To achieve single-nucleotide (nt) resolution, many techniques have been developed, such as surface-enhanced Raman spectroscopy, fluorescence resonance, circular dichroism, the electrochemical method, optical imaging, plasmonic resonance, and nanopores [8,9,10,11,12,13,14,15]. Many of these techniques require expensive instruments, which could be a constraint for both fundamental research and industrial applications. Direct observation of biological processes has also been shown using fluorescent, magnetic, or plasmonic beads, as well as dyes and other nanomaterials [16,17,18,19,20]. However, high specificity, preferably single-nt resolution, has not been preserved well in comparison with those using sophisticated apparatus.

On the other hand, various forms of force have been playing multiple roles in biological research since early on. For example, centrifuges and shakers have been basic laboratory tools to generate mechanical forces for sample preparation. Dragging force and acoustic radiation force have been used for biochemical sorting and sensing [21,22]. More recently, the field of force spectroscopy, which consists of many modern techniques, has been developed to study the mechanical properties of nucleic acids and their various functions. The techniques include atomic force spectroscopy, optical tweezers, magnetic tweezers, centrifugal force spectroscopy, acoustic force spectroscopy, as well as force-induced remnant magnetization spectroscopy and super-resolution force spectroscopy, which we invented [23,24,25,26,27,28,29,30]. However, hardly any of these force-based techniques are simple to use or cheap to build. For the scope of nucleic acid sensing, there seems to be a void in the participation of mechanical force.

Here, we show the method of force-induced visualization (FIV) with single-nt resolution. The method is based on applying mechanical force on nucleic acids labeled with magnetic microparticles. The results can be digitized to be “0” and “1” states, which can be revealed directly by the naked eye or more quantitatively using a simple absorption device. The adjustable force makes FIV suitable for many complex biomolecular applications. We demonstrate applications in distinguishing movement of ribosome on mRNA during translocation and screening drugs to reveal their sequence specificity. Further development towards multiplexed detection and resolving different samples will be discussed.

## 2. Materials and Methods

A detailed flowchart of the experimental procedure is shown in the Supplementary Information (Figure S1). The sample well, with dimensions of 4 × 2 × 1 mm^3^ (L × W × D), was coated with biotin on the bottom surface (Biotin-PEG-SVA-5000 and MPEG-SVA-5000, 1:30 ratio, Laysan Bio Inc., Arab, AL, USA). Then, 10 µL streptavidin (S888, ThermoFisher Scientific, Waltham, MA, USA) solution at 0.25 mg/mL was added to the sample well and incubated for 1 h at room temperature. After washing excess streptavidin away with the corresponding buffer solution, 10 µL aqueous solutions of nucleic acids were added into the sample well. Next, the nucleic acid solutions were washed three times with free TBS or TAM_10_ buffer, then the T_25_-conjugated M280 was added. The T_25_-conjugated magnetic particles were obtained by incubating 2 μL streptavidin-coated magnetic particles M280 (10 mg/mL, 11205D, Fisher Scientific, Hampton, NH, USA) with 30 μL biotinylated T_25_ (13.3 μM, Integrated DNA Technologies, San Diego, CA USA) for 1 h and were diluted to 24 μL after they were washed with the buffer three times. The concentration of T_25_-coated M280 was approximately 0.5 mg/mL. After 1 h of incubation, the samples were kept perpendicular for 10 min before examining magnetic particles on surfaces and taking photos. For higher mechanical forces, a centrifuge (5417R, Eppendorf) was used to apply various mechanical forces on the samples, with a speed increment of 1000 rpm (revolution per minute). The force is then calculated using *F* = *m*ω^2^*r*, in which *m* is the buoyant mass of M280, ω is the centrifuge speed, and *r* is the radius of the rotor for the centrifuge [29]. The photos were taken using an iPhone 4S (Apple, Cupertino, CA, USA). All nucleic acid oligomers were purchased from Integrated DNA Technologies (Coralville, IA, USA) and used directly. All chemicals were purchased from Sigma-Aldrich unless specified otherwise.

Precise measurements of the magnetic signal were obtained by a home-built atomic magnetometer with a sensitivity of ~150 fT√Hz [29]. The microscope was from AmScope (Irvine, CA, USA, Model T650A), with 10× objective. For transmission measurement, a common laser pointer (red beam) was used as the light source. The photodetector was DET100A (Thorlabs, Newton, NJ, USA). The neutral density filter has an attenuation of 1000 (ND = 3.0, Thorlabs).

For experiments performed in TAM_10_, the sample tube contains 18 µL TAM_10_ (1M NaCl) buffer, 3 µL biotinylated target DNA or RNA (1 µM), 6 µL ruler DNA (1 µM), and 3 µL A_25_-labeled target DNA (1 µM). The mixture was then transferred to the sample well.

The ribosome complexes were synthesized using the previous method [31]. The TAM_10_ buffer contains 20 mM tris-HCl (15568025, ThermoFisher Scientific), 30 mM NH_4_Cl (213330, Sigma Aldrich, St. Louis, MO, USA), 70 mM KCl (P3911, Sigma Aldrich), 5 mM EDTA (15575, Invitrogen, Waltham, MA, USA), 7 mM BME (2-mercaptoethanol) (M3148, Sigma Aldrich), and 10 mM MgCl_2_ (M1028, Sigma Aldrich). To form the post-MFE complex, the ribosome initiation complex programed with an mRNA coding MFES, and more, an aminoacylation solution containing 2 A/mL Phe-tRNA^Phe^ (1000 pmol/A_260_) and 10 A/mL Glu-tRNA^total^ (50 pmol/A_260_) and an EF-G/EF-Tu solution containing 3 μM EF-Tu, 2 μM EF-G, 4 mM GTP (GE272076, Sigma Aldrich), 4 mM PEP (P7127, Sigma Aldrich), and 0.02 mg/mL Pyruvate Kinase (P1506, Sigma Aldrich) were mixed and incubated at 37 °C for 30 min. After formation, the complex was purified via 1.1 M sucrose cushion. To produce the post- and pre-translocation complexes carrying MFES peptide, the post-MFE (1 μM) was incubated with or without an EF-G solution that contained 10 A/mL charged Ser-tRNA^total^ (40 pmol/A_260_), 3 μM EF-Tu, 2 μM EF-G, 4 mM GTP, 4 mM PEP, and 0.02 mg/mL Pyruvate Kinase at 37 °C for 30 min. The complexes were purified via 1.1 M sucrose cushion.

For the DNA-drug-enzyme experiments, 1 μL at 100 μM of each of the two DNA strands, 5 μL 10× NEB buffer for DpnII (R0543S, New England BioLabs, Ipswich, MA, USA) and CutSmart Buffer for EcoRI (R3101S, New England BioLabs) and HpaII (R0171S, New England BioLabs), and 41 μL autoclaved ultrapure water were mixed. The corresponding drug molecules, with various concentrations, were also added. The mixture was kept overnight. Next, 1 μL endonuclease was added into the aqueous mixture and incubated at 37 °C for 1 h, followed by inactivation at 70 °C for 20 min. Centrifugal force of 1 pN (corresponding to 500 rpm for Eppendorf 5417R) was then applied for 5 min.

For the acoustic radiation force experiments, the piezo plate was 40 × 40 × 2 mm, with resonance frequency at 1.05 MHz. The ultrasound amplitude was 0.5 V and amplified by 32 dB using an AR amplifier (75A250A). The duration was 30 s.

## 3. Results

### 3.1. Principle and Demonstration of FIV

**Figure 1:**
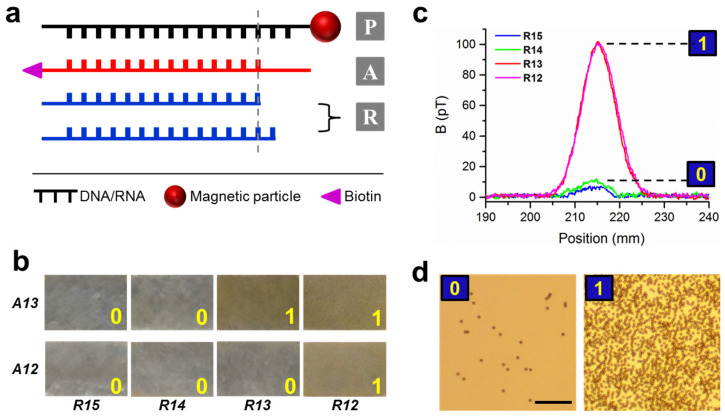
Force-induced visualization with single-nucleotide resolution. (**a**) Schematic of the method, which contains a magnetic platform P, analyte strand of unknown length A, and ruler strands R. (**b**) Photo images of using 15, 14, 13, and 12 nt rulers (R15 to R12) to measure a 13 nt target strand A13 (top row) and 12 nt strand A12 (bottom row). (**c**) Magnetic signals of the four samples shown in b for A13. (**d**) Microscopic images of the R14 (left) and R13 samples (right) for A13. Scale bar: 50 μm.

Figure 1a shows the schematic of the FIV method. A magnetically labeled DNA platform (Strand P) is designed to be complementary to the analyte strand under study (Strand A) but with one or more excessive nucleotides. Then, a series of DNA rulers (Strand R) are respectively added to the sample containing the two previous strands. Due to the competitive thermodynamic binding, two different scenarios will occur. If R is longer than A, PR hybridization will be dominant; if R is shorter than or equal to A, then PA hybridization will remain more stable than or at least the same as PR. Since A is labeled here with biotin, only PA duplex will be able to immobilize the magnetic particle on strand P to the surface. Samples with immobilized particles (denoted as state “1”) will produce stronger magnetic signals, transmit less light, and have different appearances compared to the blank samples (denoted as state “0”). All these properties can be used for distinguishing the two states.

Figure 1b shows the comparative measurements of two analyte strands: A13, which contains 13 complementary nts to strand P, and A12, with 12 complementary nts. The experiments were performed in TBS buffer (50 mM Tris-HCl (pH 7.6), 150 mM NaCl). Detailed sequence information and calculated ΔG^0^ values are in the Supplementary Information Tables S1 and S2, respectively. The rulers’ lengths went from 15, 14, 13, to 12 nts complementary to P, denoted as R15, R14, R13, and R12, respectively. The samples were placed vertically to enable gravity to remove the nonspecifically bound magnetic particles. The buoyant mass of the magnetic particles has been measured to be 4.6 × 10^−15^ kg, corresponding to a gravitational force of 0.045 pN [29]. For A13, both R15 and R14 showed no immobilized particles, and thus “0” was assigned; both R13 and R12 showed particles on the surface, which were thus assigned with “1” on the images. In contrast, for A12, R13 showed no particles because it could form more base pairs with P than A12. Therefore, single-nt resolution was achieved by using a weak mechanical force in TBS buffer. Control experiments were performed to ensure the immobilization of particles was due to DNA hybridization (Supplementary Figure S2).

The states of “0” and “1” were confirmed by measuring magnetic signals of these samples with an atomic magnetometer (Figure 1c) and with a microscope (Figure 1d). For the samples using R15 and R14 for A13, minimum magnetic signals of 7 and 10 pT, respectively, were observed. In contrast, the samples using R13 and R12 for A13 produced much higher magnetic signals of 102 pT. The results confirmed the assignments of state “0” and state “1” in the visualization detection shown in Figure 1b. The number of magnetic particles was 4.5 ± 0.1 × 10^5^ for 102 pT and 0.4 ± 0.1 × 10^5^ for 8.5 pT (average of 7 and 10 pT), based on a previous calibration [29]. The microscopic images in Figure 1d clearly revealed the difference between the two states. Particle counting yielded 4.1 ± 0.3 × 10^5^ and 0.2 ± 0.3 × 10^5^, respectively, consistent with the magnetometer results. Therefore, the two states of “0” and “1” can be easily distinguished by naked eyes, magnetometer, or microscope.

A variation of the scheme, by switching the biotin label from the analyte to the rulers, is able to detect label-free analytes (Supplementary Figure S3). In this scheme, the experimental observations of with and without particles become the opposite of the previous results: when the ruler was shorter, no particles could be immobilized because the hybridization favors the unlabeled analyte. This modified scheme can be more practical when functionalizing the analyte is not desired.

The basis of FIV is the thermodynamic equilibrium between two competitive hybridizations, which can be illustrated as

(1) PA+R⇔PR+A

The concentration ratio of the two duplexes can be approximately written as

(2) $$\left[PR]/[PA]=e^{-\Delta (\Delta G^{0})/RT}•[R]/[A\right]$$

Here, Δ(ΔG^0^) is the Gibbs free energy difference between the two duplexes, and [*R*] and [*A*] are concentrations of the ruler DNA and analyte, respectively. [*PR*] and [*PA*] are concentrations of the two duplexes. The duplex ratio governs the appearance of the sample under mechanical force: a higher concentration of PA will favor the “1” state with immobilized particles, requiring a stronger force to remove the particles. This is because the binding order between the particles and the surface increases when the density of the biomolecular pairs increases due to formation of multiple bonds. Thus, a stronger mechanical force is required to dissociate higher-order bonds [32,33]. There are two factors that determine the duplex ratio, [*R*]/[*A*] and Δ(ΔG^0^). In addition, ΔG^0^ varies under different conditions, such as ionic species and salt concentrations. Even the calculated ΔG^0^ may deviate by 1–2 kcal/mol, which will significantly affect the competition between the ruler and the analyte [34]. Therefore, an adjustable force determined by the two factors is needed to make FIV broadly applicable and to maintain single-nt resolution.

**Figure 2:**
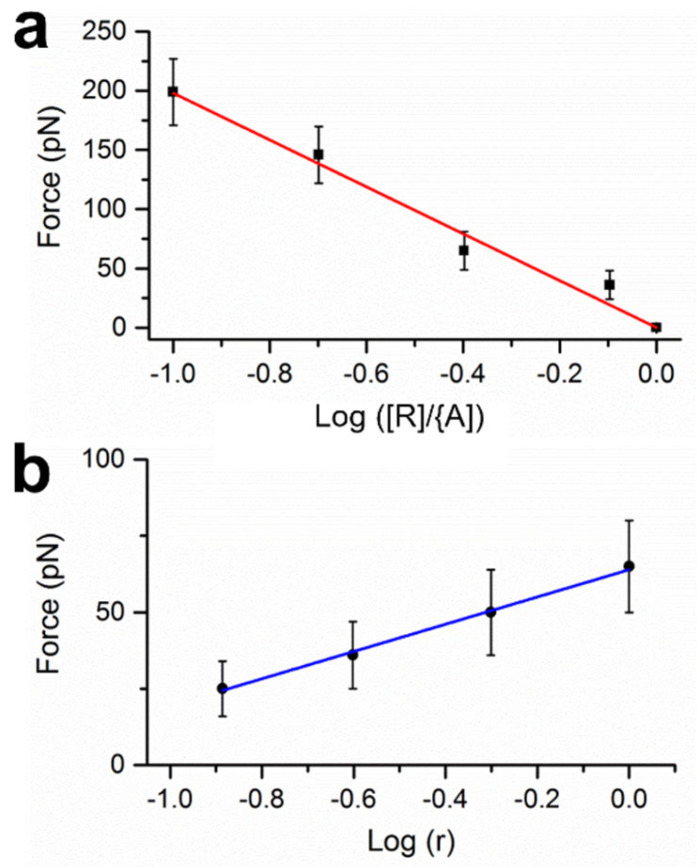
Adjustable mechanical force based on experimental conditions. (**a**) The required force vs the ratio of [*R*]/[*A*]. (**b**) The required force at various relative concentrations of TAM_10_ buffer, denoted as r.

We have quantified the influence of the two factors. In Figure 2a, the concentration of R15 was varied, while the concentrations of P and A13 were fixed at 100 nM. When R15 was at 80 nM, 40 nM, 20 nM, and 10 nM, the required force was 36, 65, 146, and 199 pN, respectively. The force–concentration correlation overcomes the situation where the analyte concentration is unknown, which is commonly encountered.

To examine the effect of complex buffer conditions on Δ(ΔG^0^), we tested the same DNA systems in TAM_10_ buffer (20 mM Tris-HCl (pH 7.5), 30 mM NH_4_Cl, 70 mM KCl, 5 mM EDTA, 7 mM 2-mercaptoethanol, 10 mM MgAc_2_) [31]. When a 65 pN centrifugal force was applied, single-nt resolution was achieved by distinguishing A13 and A12: the 0→1 transition occurred at R13 for A13 and at R12 for A12 (photos in Supplementary Figure S4a). The force value is just above the approximate dissociation force of single 13-bp DNA duplexes [31]. When the TAM_10_ buffer was diluted to 1/2, 1/4, and 1/8 in concentrations, the required force was reduced to 50, 36, and 25 pN, respectively (Figure 2b). When only gravity was applied, A13 and A12 showed the same pattern, in which both 0→1 transitions took place at R14 (Supplementary Figure S4b); the same result was obtained even when the samples were heated to 75 °C (Supplementary Figure S4c). The requirement of force indicates that R14 replacing A13 in TAM_10_ is not as efficient as in TBS, leaving a substantial amount of PA duplexes in the sample, which enables particle immobilization. In addition, using mechanical force was the only option for single-nt resolution, not the thermal approach. It is worthy of note that the applied centrifugal forces can be readily varied via adjustment of the centrifugal speeds; other well-defined mechanical forces such as acoustic radiation forces may also be applied to modulate the surface-immobilized particles [30,35].

### 3.2. Application in Probing Ribosome Translocation

**Figure 3:**
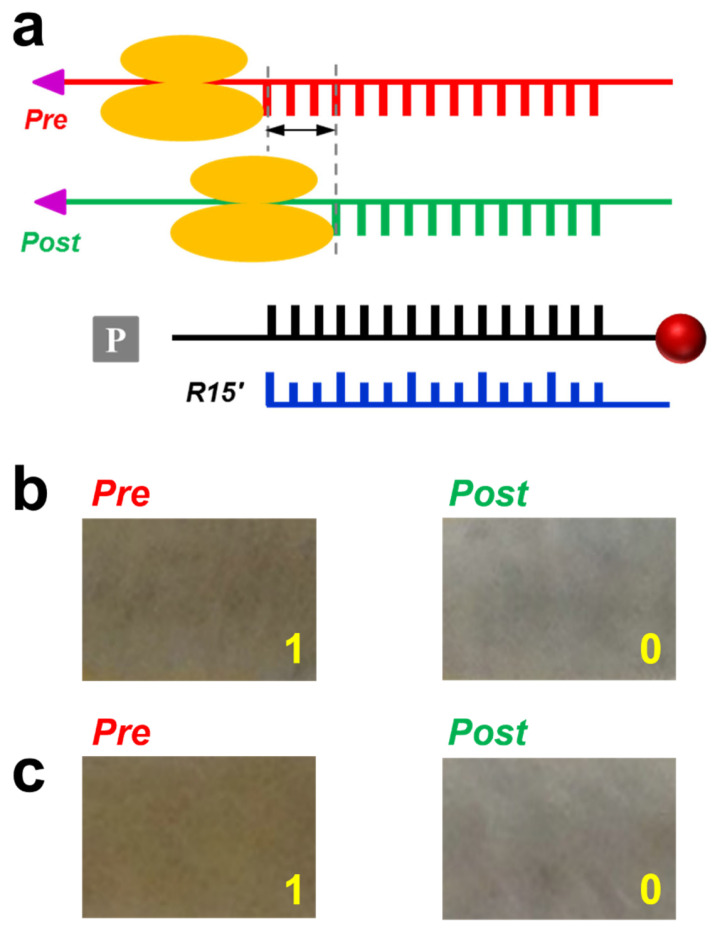
Probing the different positions of mRNA in Pre and Post ribosome complexes. (**a**) Probing scheme. (**b**) Images of Pre and Post after applying 80 pN. (**c**) Images of higher concentrations of Pre and Post after applying 230 pN.

The FIV method allows us to visually measure the lengths of nucleic acids in various biological functions without invasive sample separation or purification. The first example of application is probing the movement between the ribosome and the mRNA during ribosome translocation, a key step in keeping protein fidelity. Shown in the scheme in Figure 3, the ribosome is supposed to move three nts towards the 3′ end of the mRNA for each translocation step, from the pre-translocation complex (Pre) to the post-translocation complex (Post). The sequences of the mRNA and DNA rulers are provided in the Supplementary Information (Table S1). For Pre, the ribosome (indicated by the two orange ovals) occupies the position on the mRNA with 15 nt exposed to bind with the platform strand. If translocation took place, the ribosome would move three nts to reach the post-translocation (Post) position. Consequently, the mRNA would have only 12 nt to hybridize with the platform. The three nt difference can be revealed by ruler R15′ modulated at 80 pN because R15′ was only able to completely replace the 12-bp duplex between Post and the platform, leaving no particles on the sample surface (Figure 3b). The force value just exceeds the expected dissociation force for the 15-bp DNA/RNA duplex [31]. When the concentration of the ribosome complexes was increased by a factor of two while keeping the other parameters unchanged, the required force to distinguish Pre and Post became 230 pN (Figure 3c). This is because the ratio of [*R*]/[*A*] became half of the previous case. According to the plot in Figure 2a, the force would increase substantially. Therefore, the force value is an indicator of the analyte concentration. These results provide an efficient and simple way to estimate the quality of the biomolecular analyte, which is cheaper and safer than the conventional radioactivity assays.

### 3.3. Application in Sequence-Specific Drug Screening

**Figure 4:**
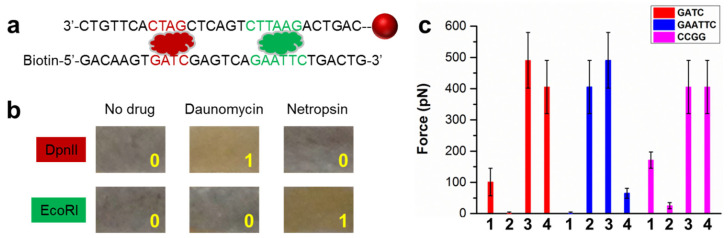
Visualization of drug binding specificity. (**a**) Scheme for FIV. The DNA duplex contains two specific sites for endonucleases, indicated in dark red and green, respectively. (**b**) Photos of the results after applying 1 pN force. (**c**) Screening of four drugs against three DNA sequences. 1: daunomycin; 2: netropsin; 3: doxorubicin; 4: epirubicin.

Since the force amplitude depends on the thermodynamic stability of the nucleic acid duplexes, the FIV method can be used for sequence-specific studies of drug molecules with the assistance of restriction enzymes. Here, the competing equilibrium is between drug-bound DNA duplex and free duplex. Only the former will lead to particle immobilization because the latter will be cleaved by the enzyme, as shown in the scheme in Figure 4a. One of the DNA strands is labeled with biotin, while the other is conjugated with a magnetic particle. If a drug molecule binds at the enzyme-specific site on the DNA duplex, the drug will block the cleavage. Consequently, the magnetic particles will be conjugated to the surface, leading to state “1”.

The shown DNA duplex contains two endonuclease sites: GATC for DpnII and GAATTC for EcoRI [36,37]. Two drug molecules were investigated: daunomycin and netropsin [38,39]. Their molecular structures are shown in Supplementary Figure S5. The results are shown in Figure 4b. In the absence of a drug, the duplex can be cleaved by either nuclease such that there are no immobilized particles (left two). In the case of daunomycin, it binds with the specific site of DpnII and blocks the enzyme binding [40]. Therefore, the DNA duplexes remained intact, allowing the magnetic particles to be immobilized on the surface. Therefore, the sample showed state “1” (top middle). However, daunomycin does not specifically bind the GAATTC, the interacting site of EcoRI, so the DNA duplex was cleaved. No particles were present in the sample (bottom middle). Similarly, netropsin only binds with the AATT site, overlapping with the EcoRI binding site [39]. Thus, particles were present in the sample with EcoRI but absent with DpnII.

To further quantify the strength of drug binding, we increased the force such that the majority of the particles were dissociated. The required force thus represented the relative binding strength of the drug on the particular sequence. Four drug molecules against three sequences were studied. The CCGG-specific enzyme was HpaII [41]. The results in Figure 4c showed doxorubicin bound strongly with all three sequences, with high and almost equal forces. Epirubicin, which differs from doxorubicin by only the chirality of a carbon atom, bound with GAATTC much more weakly than the other two sequences. Interestingly, epirubicin has been found to be less toxic than doxorubicin [42,43]. The results also showed the CCGG binding site for daunomycin. Compared to doxorubicin, daunomycin binding is generally much weaker, which can be attributed to the extra OH group that doxorubicin contains. The fairly weak binding of daunomycin onto CCGG and strong binding of doxorubicin onto GAATTC were confirmed by using force spectroscopy (Supplementary Figure S6).

### 3.4. A device for Recording and Quantification

**Figure 5:**
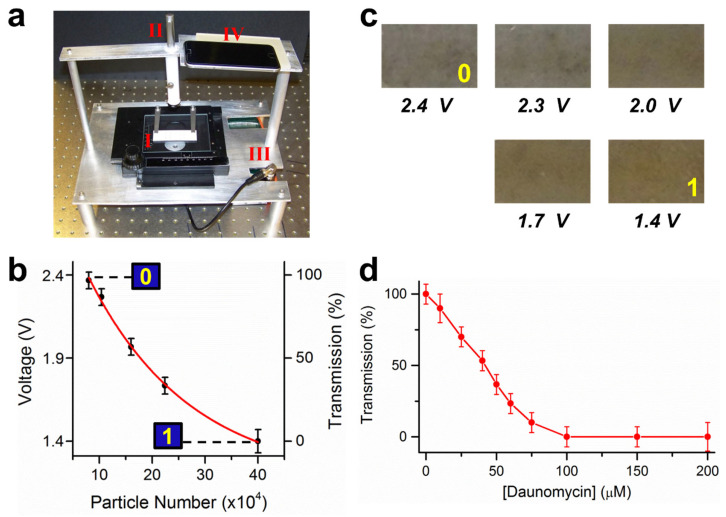
A dual-purpose device for recording and quantification. (**a**) Photo of the device. I: sample holder; II: laser pointer; III: voltmeter connected with a photodetector; IV: holder for a smart phone. (**b**) Correlation between the voltage measured by the photodetector and the number of magnetic particles in the sample well. The fitting was a single-exponential curve. (**c**) The corresponding photos of the samples. (**d**) Determining the binding constant of daunomycin using transmission measurements.

To facilitate the recording and quantification of the FIV experiments, a cheap and portable device has been constructed, which cost approximately $300 and could cost much less (Figure 5). It contained a sample holder for mounting a multiplexed sample plate, a laser pointer as the light source, and a photodetector with a neutral density filter located beneath the sample (not visible in the photo) and connected with a voltmeter. In addition, the photos of the sample can be taken by a smart phone, which is held in place by a holder. A light beam with a diameter of 2 mm was obtained after the laser light passed through an iris diaphragm. In our study, a voltage of 2.4 V (error bar ± 0.1 V) or higher corresponded to >4.0 ± 0.5 × 10^5^ particles, which was designated as state “0” (high transmission); and 1.4 V or lower corresponded to <0.8 ± 0.5 × 10^5^ particles, which represented state “1” (low transmission). The measurements agreed with the “0” and “1” states that we defined using magnetic and microscopic measurements shown in Figure 1.

More precise quantification can be obtained by correlating the number of magnetic particles with the voltage measurements between the range of 1.4–2.4 V. The fitting curve in Figure 5b shows that the quantification curve can be established, from which the number of magnetic particles, hence the quantity of the unknown strand, can be determined from the voltages measured. The sample images corresponding to the data points are shown in Figure 5c. By using the transmission measurement, states between “0” and “1” can be quantified.

To show the advantage of quantification, we measured the binding constant of daunomycin. The profile in Figure 5d showed saturation at 75 μM, at which the transmission is the same as those of high concentrations within the experimental error. This value is similar to previous spectroscopic and magnetic measurements [40,44]. This result further validates the quantification capability of the device.

### 3.5. Multiplexed Detection Using Acoustic Radiation Force

To improve the applicability of FIV and its device, multiplexed detection is desired. This would require a different force field than the centrifugal force, because it is difficult to fit a large sample plate into a centrifuge. We have recently shown that precise acoustic radiation force can be generated on magnetic microparticles by ultrasound generation, which is also commonly used for particle manipulation [45]. However, it has not been used for multiple samples on a large plate.

**Figure 6:**
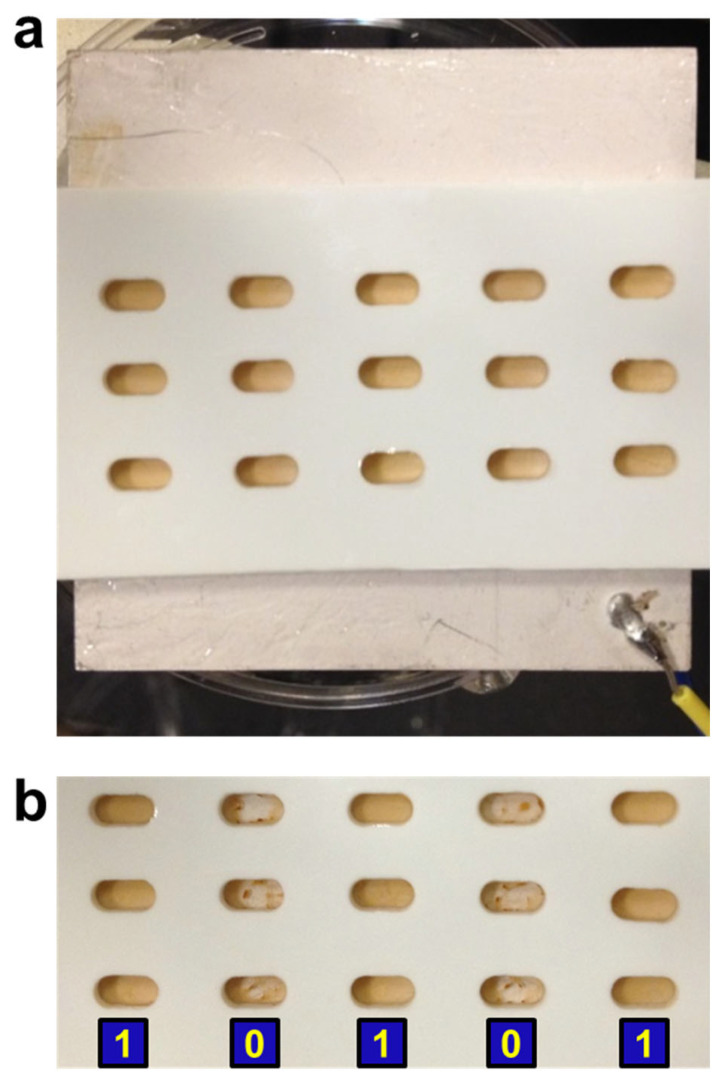
Multiplexed detection using acoustic radiation force. (**a**) A 15-sample plate placed on a piezo prior to applying ultrasound. (**b**) The sample plate after applying ultrasound. Each sample well was 4 mm long and 2 mm wide.

The result of our attempt is shown in Figure 6. The sample plate contained fifteen sample wells and was placed on a large piezo piece. Prior to ultrasound application, all the samples had the same appearance because the magnetic particles sink to the bottom surface of each well, regardless of specific and nonspecific binding. In other words, they were all at state “1” (Figure 6a). The conditions are that column 1 contains daunomycin and DpnII, column 2 contains netropsin and DpnII, column 3 is DNA duplex only, column 4 contains daunomycin and EcoRI, and column 5 contains netropsin and EcoRI. After applying ultrasound, the samples in columns 2 and 4 showed state “0”, and the other three columns remained at state “1” (Figure 6b). This is because the DNA duplexes in columns 2 and 4 were cleaved by their respective endonucleases, whereas the cleavage was blocked by the corresponding drugs in columns 1 and 5. Column 3 is the control without drug or enzyme. The results are consistent with the results of individual samples shown in Figure 4b. It is worth mentioning that all fifteen samples were successful, showing the robustness of our FIV method. When gravity is sufficient, the sample plate can be simply placed vertically for the free particles to fall. An example is shown in Supplementary Figure S7.

## 4. Discussion

The force amplitude to resolve nucleic acids with single-nt resolution is determined by two independent factors: the ratio of DNA ruler to the unknown analyte and the difference in ΔG of the two competing binding processes. For many potential applications, one of the parameters is well defined, for example, the analyte’s sequence or buffer condition. In these cases, the force amplitude will be a useful parameter to determine, for example, the analyte’s concentration. In other words, the FIV technique can be analogous to a UV spectrometer or other similar analytical devices. When neither is known, systematic titrations need to be carried out with a series of DNA rulers to reveal both the sequence and concentration of the nucleic acid under the specific experimental conditions. Compared to other visual or colorimetric detection techniques, our approach is able to precisely determine the length of nucleic acids without amplification. However, the data presented here are limited to the scope of nucleic acid and do not yet include the potentially broader applications in protein-specific detections.

An interesting scenario is when there is more than one population of a specific analyte. For instance, frameshifting is a common mechanism used by viruses in which the ribosome translocates different numbers of nucleotides on the mRNA [46]. For this more challenging case, a simple “1” vs. “0” will no longer be valid. Instead, quantification should be carried out by using the absorption measurements with the device shown in Figure 5. More effort is needed to improve the capability of resolving a second population with low percentages, as do any sensing techniques at their infant stage.

## 5. Conclusions

We have established the FIV method, which uses an adjustable mechanical force to measure the precise lengths and function sites of nucleic acids during different biomolecular processes. Single-nt resolution has been achieved by clearly distinguishable states of “0” and “1”. Mechanical force modulation is a unique approach that cannot be replaced by thermal activation. Equipped with a portable device in conjunction with a smart phone, recording and quantification can be achieved. Multiplexed detection is demonstrated using acoustic radiation force. Combining high resolution, versatile quantification, and simple implementation, our FIV method will find broad applications in biochemical research and industry.

## 6. Patents

A patent application is pending, under application number US 62/517,694.

## Data Availability

Data is available upon request to the corresponding author.

## Supplementary Materials

The following supporting information can be downloaded at https://www.mdpi.com/article/10.3390/s23187762/s1, which includes two supplementary tables and seven supplementary figures. Table S1: Sequences of the DNAs and RNAs; Table S2: DG° of duplexes; Figure S1: Flowchart of the experimental procedure; Figure S2: Control experiments to confirm particle immobilization; Figure S3: A variation of FIV scheme for detecting label-free analytes; Figure S4: Results in TAM_10_ buffer with different forces and at elevated temperature; Figure S5: Structures of the four drug molecules; Figure S6: Force spectra of daunomycin and doxorubicin binding; Figure S7: Multiplexed detection using only gravity.
