# Supplementary material for: Force-Induced Visualization of Nucleic Acid Functions with Single-Nucleotide Resolution
Source: Sensors (Basel). 2023 Sep 8;23(18):7762. doi: 10.3390/s23187762 (PMC10536483; doi:10.3390/s23187762)
Supplement: Supplementary file 1 [file sensors-23-07762-s001.zip › sensors-2577327-supplementary.pdf]

Supplementary Information

Force-induced visualization of nucleic acid functions with single-nucleotide resolution

Table of Contents:

1. Sequences of the DNAs and RNAs, with Table S1; and ΔGº of duplexes, with Table S2;
2. Flowchart of the experimental procedure, with Figure S1;
3. Control experiments to confirm particle immobilization, with Figure S2;
4. A variation of FIV scheme for detecting label-free analytes, with Figure S3;
5. Results in TAM_{10} buffer with different forces and at elevated temperature, with Figure S4;
6. Structures of the four drug molecules, with Figure S5;
7. Force spectra of daunomycin and doxorubicin binding, with Figure S6;
8. Multiplexed detection using only gravity, with Figure S7.

1. Sequences of DNAs and RNAs and ΔG° of their duplexes

Table S1. Sequences of the DNAs and RNAs used in this work.

Name	Sequence (from 5′ to 3′)
A13	Bio/CAG ACT GAC TCG A[a]
A12	Bio/CAG ACT GAC TCG
P15	A_{25}/GAT CGA GTC AGT CTG[b]
R15	CAG ACT GAC TCG ATC
R14	CAG ACT GAC TCG AT
R13	CAG ACT GAC TCG A
R12	CAG ACT GAC TCG
R11	CAG ACT GAC TC
mRNA (Pre)	Bio/C AAC UGU UAA UUA AAU UAA AUU AAA AAG GAA AUA AAA AUG UUU GAA AGU AAG UA**C GUA AAU CUA CUG CUG AAC UC**[c]
mRNA (Post)	Bio/C AAC UGU UAA UUA AAU UAA AUU AAA AAG GAA AUA AAA AUG UUU GAA AGU AAG UAC GU**A AAU CUA CUG CUG AAC UC**[c]
R15′	A_{25}/CT CAA GAG CAG TAG ATT TAC G[d]
$CCGG_1$	Bio/GAC AAG TGA CCG GGT CAG TCT GTA ATA A
$CCGG_2$	Bio/CAG ACT GAC CCG GTC ACT TGT C

[a]Bio: biotin labeled; [b]Functionalized with 25 units of A.

[c]Bold indicating the ribosome-uncovered nucleotides; underscored indicating the segment for probing.

[d]Underscored indicating the complementary nucleotides to the mRNA.

Table S2. Δ*G*° of the duplexes between the ruler strands and the platform strand.[e]

Ruler	Platform	Δ*G*° (kcal/mol)
R11		-11.9
R12		-14.3
R13	P15	-15.5
R14		-16.5
R15		-17.2

[e]Calculated using DNAmelt software (available online). Reference: *Nucleic Acids Res.* **33**, W577, (2005). Conditions: 37 °C, [DNA] = 100 nM, $[Na^+]$ = 1 M, and $[Mg^{2+}]$ = 0 M.

2. Flowchart of the experimental procedure

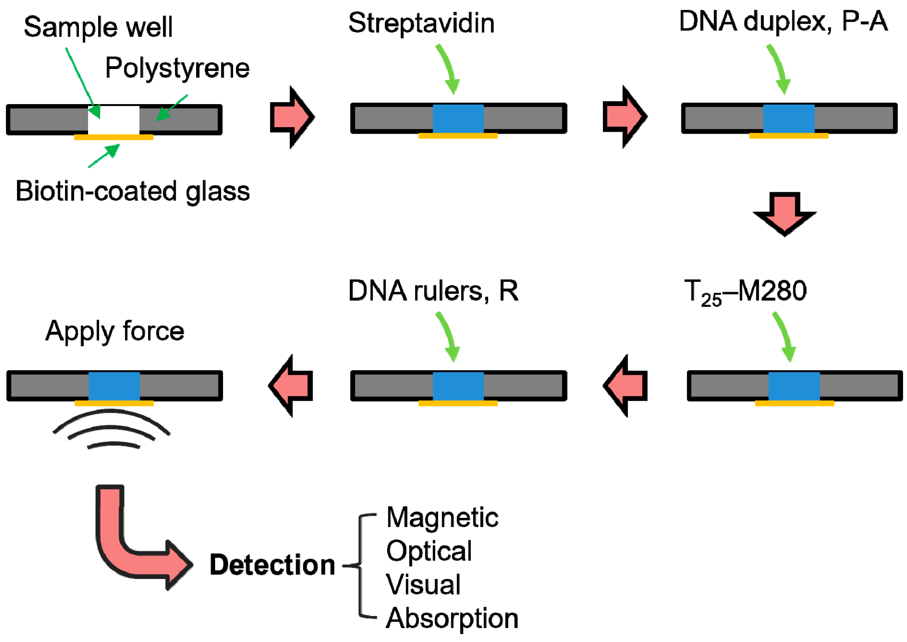

Figure S1. Flowchart of the experimental procedure.

3. Control experiments to confirm particle immobilization

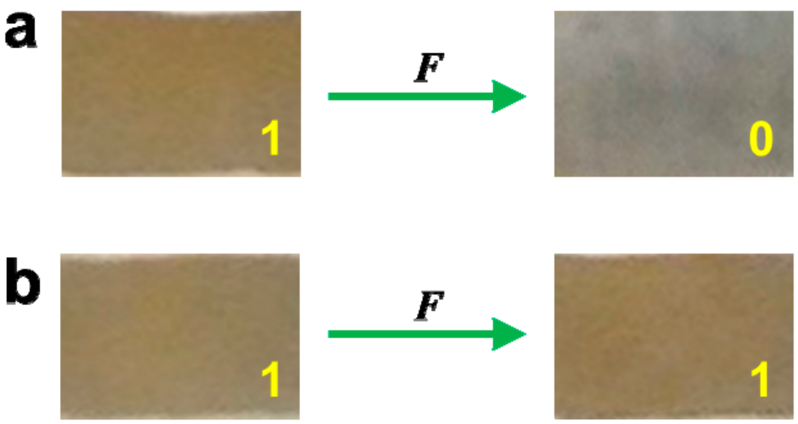

Figure S2. Confirmation of particles immobilized only via DNA duplexes. **a**. Photos of a sample containing single DNA strands before and after applying gravity. Particle immobilization cannot be achieved because only one end of the DNA was labeled, which has been used to bind with the particles. Therefore, nearly all particles fall off the surface after applying the gravity force by placing the sample vertically. **b**. Photos of a sample containing DNA duplexes before and after applying gravity. Particle immobilization can now be achieved through DNA duplexes: one strand of the duplex using its biotin end to bind with the streptavidin on the surface, the other strand binding using its A_{25} end to bind with the magnetic particles conjugated with T_{25}.

4. **A variation of FIV scheme for detecting label-free analytes**

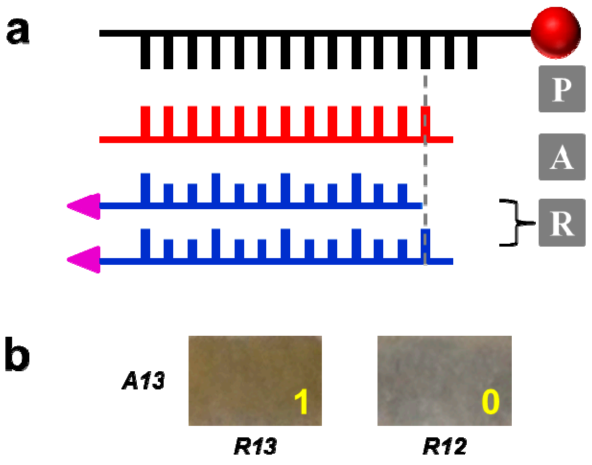

Figure S3. Modified FIV scheme and results for label-free analytes. **a.** Scheme, in which Strand A is not labeled, and Strand R's are labeled with biotin. **b.** Photos of using R13 and R12 to measure unlabeled A13, respectively.

5. **Results in TAM_{10} buffer with different forces and at elevated temperature**

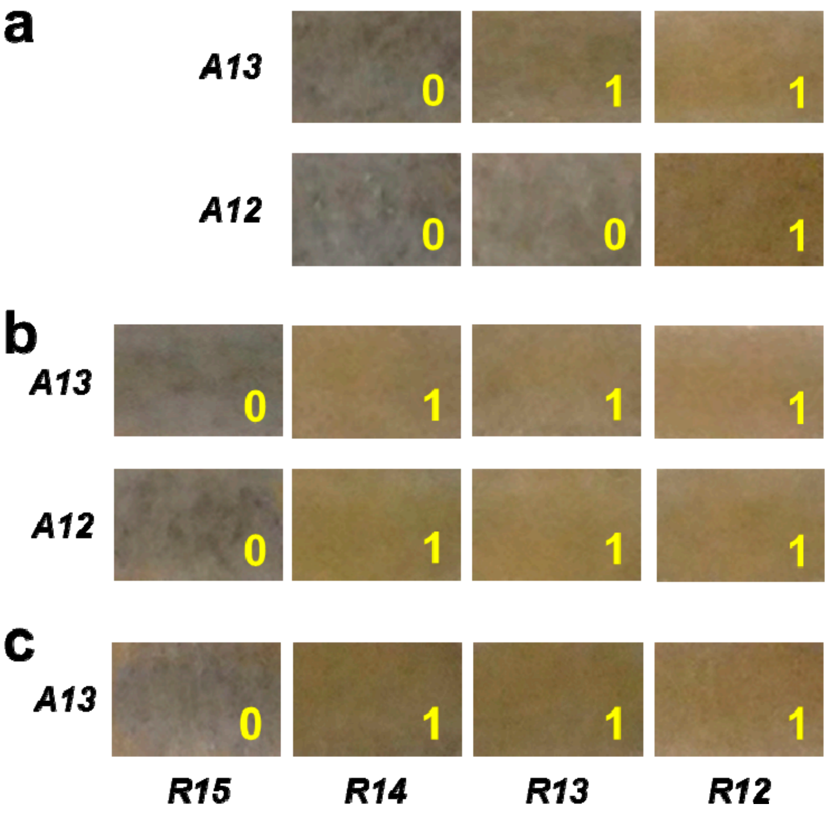

Figure S4. Results of different approaches in TAM_{10} buffer. **a.** Using 65 pN centrifugal force. **b.** Using only gravity force. **c.** At 75 °C.

6. Structures of the four drug molecules

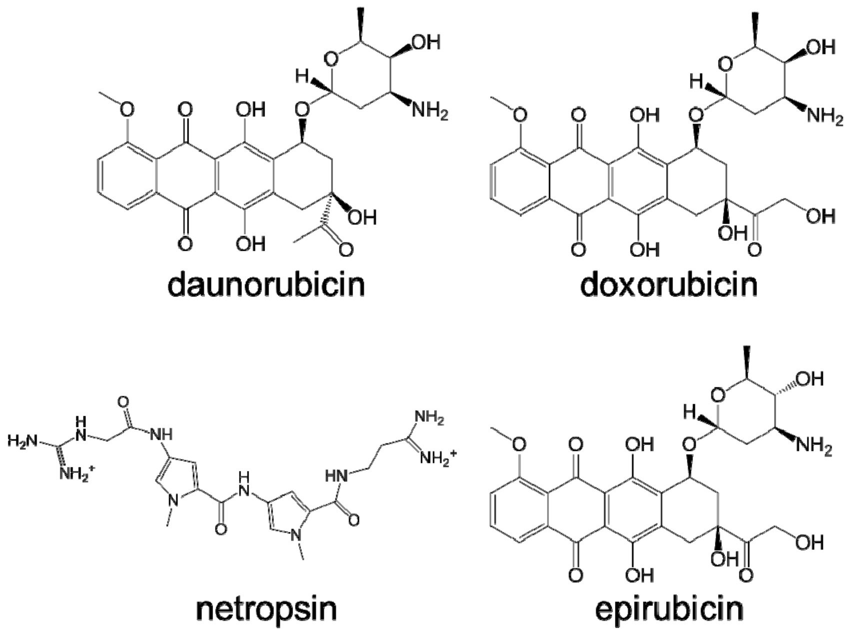

Figure S5. Structures of the drug molecules.

7. Force spectroscopy of daunomycin and doxorubicin binding

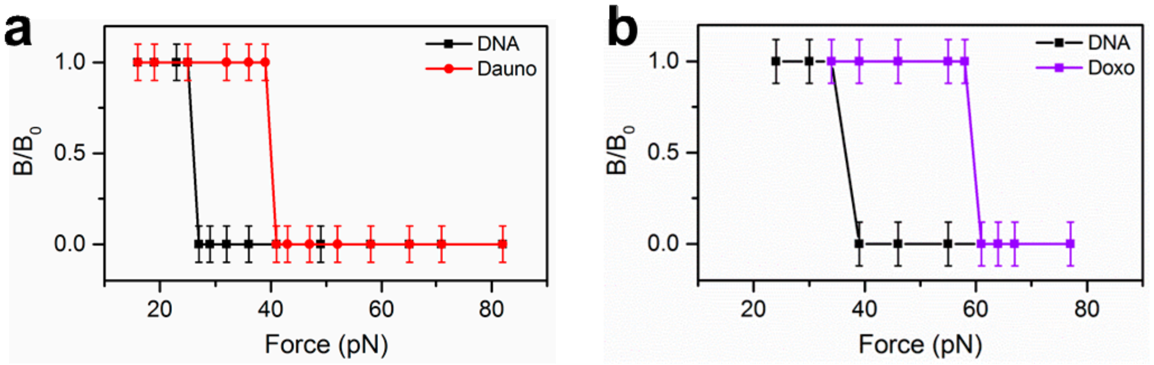

Figure S6. Force spectra obtained by force-induced remnant magnetization spectroscopy (FIRMS). **a.** Daunomycin binding with DNA. Dauno: daunomycin. The force increase was 14 pN. The DNA duplex was formed between 5′-Biotin-GGA AAC CA AAG G and 5′-Biotin-CCT TTG GTT TCC. **b.** Doxorubicin binding with DNA. Doxo: doxorubicin. The force increase was 24 pN, much greater than the effect of daunomycin binding. The DNA duplex was formed between 5′-Biotin-TCA GAA TTC TGA and its complementary sequence. The protocol for FIRMS was reported in a previous publication (*ACS Chem. Biol.* **12**, 1629-1635 (2017).).

8. Multiplexed detection using only gravity

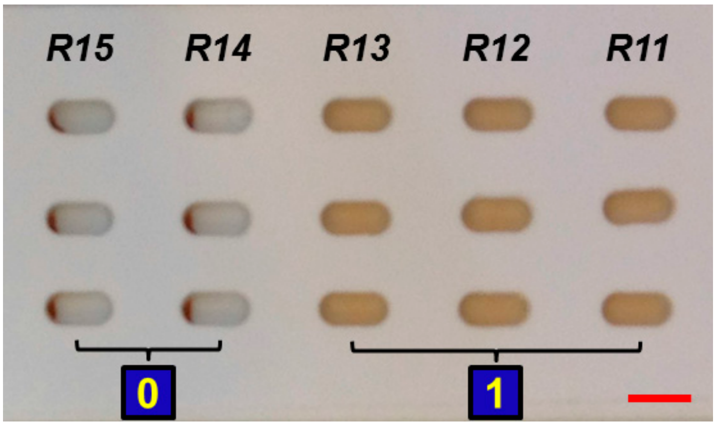

Figure S7. Multiplexed detection of FIV using only gravity. The analyte strand was A13 and the rulers were R15-R11. The experiments were repeated three times, displayed as three rows. Scale bar: 4 mm.
